# Efficacy and safety of immunotherapy in pediatric malignant brain tumors: a systematic review and exploratory meta-analysis

**DOI:** 10.3389/fonc.2026.1932372

**Published:** 2026-09-25

**Authors:** Tomasz Tykocki

**Affiliations:** 1 Department of Paediatric Neurosurgery, Children’s Hospital named after Prof. Dr. med. Jan Bogdanowicz in Warsaw, Warsaw, Poland; 2 Faculty of Medicine, Lazarski Medical University of Warsaw, Warsaw, Poland

**Keywords:** CAR-T, immunotherapy, neurotoxicity, overall survival, pediatric brain tumors

## Abstract

**Background:**

Immunotherapy for pediatric brain tumors has expanded rapidly, yet its effects on survival and neurologic safety remain unclear. A systematic review and meta-analysis was conducted to quantify efficacy and toxicity across immunotherapy modalities.

**Methods:**

Following PRISMA 2020 guidelines, prospective pediatric immunotherapy studies were identified. Random-effects meta-analyses were performed using vaccine-based immunotherapy as the reference. Primary endpoints were overall survival (OS) and 12-month overall survival (OS12); grade ≥3 neurotoxicity was the primary safety outcome. Meta-regression and sensitivity analyses explored modality-specific effects.

**Results:**

Twenty studies were included. Median OS ranged from 3.6 months in heavily pretreated recurrent cohorts to 17.8 months in selected intratumoral oncolytic virus trials, while OS12 ranged from 8–13% to 75%. Compared with vaccines, non-vaccine immunotherapy did not improve OS (pooled HR 1.14, 95% CI 0.99–1.31; I² 43%) or OS12 (HR 1.13, 95% CI 0.95–1.34; I² 65%). Subgroup analyses showed comparable OS for CAR-T therapies (OS HR 1.09, 95% CI 0.89–1.34) and checkpoint/systemic therapies (OS HR 1.14, 95% CI 0.96–1.36), or oncolytic/viral therapies (OS HR 1.51, 95% CI 0.81–2.84). Severe neurotoxicity differed substantially by modality. No grade ≥3 neurotoxicity occurred in vaccine studies. In contrast, pooled non-vaccine therapies showed a markedly increased risk (OR 19.66, 95% CI 6.78–57.02; I² 60%). CAR-T therapies (OR 57.95, 95% CI 16.15–207.98) and oncolytic/viral approaches (OR 69.41, 95% CI 17.00–283.33) had the highest risk, whereas checkpoint/systemic therapy showed a lower, non-significant increase (OR 2.26, 95% CI 0.77–6.60).

**Conclusions:**

Across pediatric immunotherapy trials, survival outcomes were comparable to vaccine-based approaches, but neurotoxicity varied markedly by modality. Cellular and oncolytic therapies carry a substantial neurologic risk that must be balanced against uncertain survival benefit in future trial designs.

## Introduction

Malignant pediatric brain tumors remain among the most lethal childhood cancers, with diffuse midline glioma (DMG), high-grade glioma (HGG), and recurrent embryonal tumors demonstrating persistently poor outcomes despite maximal multimodal therapy (1). Conventional approaches—including surgery, radiotherapy, and cytotoxic chemotherapy—have reached a therapeutic plateau, particularly in tumors characterized by intrinsic radioresistance, diffuse infiltration, and profound immunosuppression within the central nervous system (CNS) microenvironment (2, 3).

In recent years, immunotherapy has emerged as a promising strategy in pediatric neuro-oncology, driven by advances in tumor immunobiology and successful translation of immune-based treatments in systemic malignancies. Multiple immunotherapeutic platforms have entered early-phase clinical testing in children with CNS tumors, including immune checkpoint inhibitors (4, 5),peptide and dendritic-cell vaccines (6, 7), oncolytic and viral therapies (8, 9), and, most recently, engineered cellular therapies such as chimeric antigen receptor T (CAR-T) cells and natural killer (NK) cells (10, 11).

Unlike systemic cancers, the unique anatomical and immunological features of the CNS impose substantial constraints on immunotherapy delivery and safety. The blood–brain barrier, limited lymphatic drainage, and risk of immune-mediated neurotoxicity have prompted the exploration of alternative drug administration routes, including intratumoral, intraventricular, intracerebroventricular, and convection-enhanced delivery, in addition to conventional intravenous or systemic administration (12, 13). These route-specific strategies aim to enhance tumor exposure while minimizing systemic toxicity, yet may themselves confer distinct risks of neurologic adverse events.

Early-phase pediatric immunotherapy trials have reported heterogeneous efficacy signals, with sporadic radiographic responses and occasional prolonged survival in selected patients, particularly in oncolytic viral therapy and CAR-T platforms (9, 11). However, severe neurologic toxicities—including immune effector cell-associated neurotoxicity syndrome (ICANS), cerebral edema, seizures, and treatment-related encephalopathy—have emerged as a critical safety concern, particularly for locoregional and cellular approaches (10, 12).

To date, no comprehensive quantitative synthesis has systematically compared survival outcomes and neurotoxicity risk across pediatric brain tumor immunotherapy platforms while accounting for route of administration. Prior reviews have largely focused on narrative summaries or modality-specific experiences, without formal meta-analytic evaluation of efficacy–toxicity trade-offs (14, 15). Given the expanding clinical use of intratumoral, intraventricular, and systemic immunotherapies in children, a rigorous comparative framework is urgently needed to inform trial design, patient selection, and risk stratification.

Therefore, a systematic review and random-effects meta-analysis of clinical studies was performed to evaluate immunotherapy in pediatric malignant brain tumors. The aim of this analysis is to (i) quantify overall survival (OS) and 12-month OS across immunotherapy modalities, (ii) assess the incidence and comparative risk of grade ≥ 3 neurotoxicity.

## Methods

### Search strategy and study selection

This systematic review and meta-analysis was conducted in accordance with the PRISMA 2020 guidelines. A comprehensive literature search was performed in PubMed/MEDLINE, Embase, and Scopus from database inception through December 31, 2025. The search strategy combined controlled vocabulary and free-text terms related to pediatric brain tumors and immunotherapy. The complete database-specific search strategies, including all search terms, Boolean operators, and combinations used for each database, are provided in the **Supplementary Methods** to permit reproducibility.

Eligible studies met the following criteria: (i) prospective clinical studies or trials; (ii) pediatric population (≤18 years; studies including adolescents/young adults were included if pediatric outcomes were extractable); (iii) malignant CNS tumors; (iv) treatment with any form of immunotherapy; and (v) reporting at least one survival or safety outcome of interest. Reviews, preclinical studies, case reports, and studies without extractable clinical outcomes were excluded.

Trials enrolling mixed pediatric, adolescent/young-adult, and adult populations were eligible when outcomes for patients aged ≤18 years could be separately extracted or reliably reconstructed from Kaplan–Meier curves or tabular data. Studies were excluded when pediatric outcomes could not be distinguished from those of the older population.

The systematic search and eligibility assessment were restricted to studies identified within the prespecified search period ending December 31, 2025. Publications appearing after this cutoff were not eligible for inclusion in the quantitative synthesis; later publications cited in the Discussion were used solely as contextual literaturę.

A single reviewer conducted title and abstract screening with the aid of computer-assisted screening tools, followed by full-text assessment.

### Outcomes and endpoint definitions

- Overall Survival (OS): defined as time from first immunotherapy administration to death from any cause. When hazard ratios (HRs) were not explicitly reported, they were estimated from published Kaplan–Meier curves using established reconstruction methods.
- 12-month Overall Survival (OS12): defined as the probability of survival at 12 months following treatment initiation. OS12 was extracted directly when reported or derived from Kaplan–Meier curves.
- Grade ≥3 neurotoxicity included severe treatment-related neurologic adverse events such as seizures, encephalopathy, cerebral edema, or ICANS (Immune effector Cell-Associated Neurotoxicity Syndrome) -like syndromes, harmonized across reporting frameworks and conservatively mapped to severe neurologic toxicity when formal CTCAE grading was unavailable. Grade ≥3 neurotoxicity was selected as a CNS-specific safety endpoint because severe neurologic complications are of particular relevance in brain tumor immunotherapy. This endpoint was not intended to represent overall treatment toxicity. Other clinically relevant adverse events differ substantially between therapeutic platforms and include cytokine release syndrome and hematologic toxicity with cellular therapies, systemic immune-related adverse events with checkpoint inhibition, and inflammatory or procedure-related complications with locoregional therapies.

### Data extraction

Data were extracted by a single investigator using a predefined standardized form and included: study design, tumor type, immunotherapy modality, route of administration intravenous/systemic; intratumoral/convection-enhance delivery; intraventricular/intracerebroventricular/locoregional; peripheral vaccination), sample size, follow-up, median OS, HRs and confidence intervals, OS12, and neurotoxicity events.

For studies reporting multiple treatment arms with distinct administration routes (e.g., intravenous vs intracranial CAR-T), arms were treated as independent cohorts without double-counting patients.

The Dunkel et al. trial was analyzed as two independent cohorts for survival outcomes (OS | OS12), reflecting distinct treatment regimens (nivolumab alone and nivolumab plus ipilimumab) and non-overlapping patient populations. In contrast, grade ≥3 neurotoxicity was analyzed as a single combined cohort.

### Meta-analysis methods

#### Effect measures

- Survival outcomes (OS and OS12) were summarized using HRs on the log scale.
- Neurotoxicity was summarized using ORs comparing each modality or route to vaccine-based immunotherapy as the reference group.

#### Statistical model

All pooled analyses were performed using random-effects models (DerSimonian–Laird estimator) to account for between-study heterogeneity. Effect estimates were pooled on the logarithmic scale and back-transformed for presentation. Heterogeneity was quantified using I² and τ² statistics.

#### Estimation of hazard ratios for survival outcomes

HRs for overall survival (OS) and 12-month overall survival (OS12) were extracted directly when reported. When unavailable, HRs were estimated from published Kaplan–Meier curves using established reconstruction methods described by Parmar et al. (16) and Tierney et al. (17). Survival curves were digitized, and time-to-event data were reconstructed to derive log-HRs and corresponding variances.

OS12 was extracted directly at the 12-month landmark or derived from reconstructed survival curves when not explicitly reported. All HRs were analyzed on the logarithmic scale, with standard errors obtained from reconstructed variance estimates. These methods are standard for meta-analysis of survival data in the absence of individual patient data and were applied consistently across studies.

Methods for reconstruction of published survival data were used to recover time-to-event information where required; they do not eliminate confounding arising from cross-study differences in baseline prognosis, patient selection, or treatment setting. Consequently, reconstructed and pooled survival estimates were interpreted descriptively and exploratorily.

### Subgroup and route-based analyses

Pre-specified subgroup analyses were conducted according to:

1. Immunotherapy modality: CAR-T/engineered cellular therapy; checkpoint/systemic immunotherapy; oncolytic/viral therapy; vaccines.
2. Route of administration:
3. Intravenous/systemic
4. Intratumoral/convection-enhanced delivery
5. Intraventricular/intracerebroventricular/locoregional
6. Peripheral vaccination

Subgroup-specific pooled HRs (OS, OS12) and ORs (neurotoxicity) were calculated using random-effects models.

### Reference group selection and interpretation of HRs and ORs, 

Vaccine-based immunotherapy was selected *a priori* as a pragmatic reference category for exploratory cross-study comparisons and not as a therapeutic standard-of-care or randomized control group. This choice provided a common analytical benchmark across heterogeneous immunotherapy studies. Because the underlying studies were predominantly non-randomized and differed substantially in tumor type, disease setting, prior therapy, and treatment delivery, relative estimates against the vaccine category should be interpreted exclusively as exploratory indirect benchmarks and not as causal treatment effects or estimates of therapeutic superiority or inferiority.

### Meta-regression analysis

Random-effects meta-regression was performed to explore whether heterogeneity in outcomes was explained by prespecified moderators, including:

- Immunotherapy modality
- Route of administration
- Trial phase (early-phase vs phase III)
- Disease setting (newly diagnosed vs recurrent)

Regression coefficients were estimated on the log-effect scale and reported with 95% confidence intervals.

Additional clinically relevant moderators considered included tumor type, age, and prior treatment exposure. These variables were not incorporated into the multivariable meta-regression because reporting was incomplete across studies and the limited number of available cohorts would result in substantial model overfitting. Disease setting (newly diagnosed versus recurrent) was retained as a prespecified study-level moderator.

### Sensitivity analyses of meta-regression

Model robustness was evaluated using leave-one-predictor-out sensitivity analyses, whereby each moderator was sequentially removed and changes in residual heterogeneity (Δτ²) were quantified. The proportion of explained heterogeneity was summarized using pseudo-R² statistics.

This approach was used to identify dominant drivers of heterogeneity and to distinguish biologically meaningful moderators from redundant covariates.

### Risk of bias and small-study effects

Risk of bias was assessed using the ROBINS-I tool, with evaluation of bias due to confounding, participant selection, classification of interventions, deviations from intended interventions, missing data, outcome measurement, and selection of reported results. Small-study effects and potential publication bias were explored using funnel plots and Egger’s regression test, restricted to studies with estimable standard errors. Studies with zero events or non-estimable variances were excluded from funnel-plot diagnostics but retained in pooled analyses.

Small-study effects and publication bias were explored using funnel plots and Egger’s regression test, restricted to studies with estimable standard errors. Studies with zero events or non-estimable variances were excluded from funnel diagnostics but retained in pooled analyses.

### Data independence and double counting

To avoid double counting, studies reporting multiple cohorts or treatment arms were examined in detail. Separate cohorts (e.g., different routes of administration) were included as independent units only when non-overlapping patient populations and distinct outcome reporting were confirmed. When potential overlap could not be excluded, the most complete dataset was retained. No study contributed more than once to any pooled estimate for a given outcome.

### Statistical analysis

Statistical analyses were performed using Review Manager (RevMan; Cochrane) and Python version 3.14.6. All statistical tests were two-sided, and a p-value <0.05 was considered statistically significant.

## Results

A systematic literature search was performed in PubMed, Embase, and the Cochrane Central Register of Controlled Trials from database inception to the final search date. The search identified 1,312 records, of which 416 duplicates were removed, leaving 896 studies for title and abstract screening. After exclusion of ineligible reports, 143 full-text articles were assessed. Ultimately, 20 studies met the predefined inclusion criteria and were included in the final synthesis (**Figure 1**). These studies encompassed CAR-T and engineered cellular therapies, checkpoint and systemic immunotherapy, oncolytic or viral approaches, and vaccine-based immunotherapy, with substantial variability in tumor type, disease setting, and route of administration. Characteristics of all included studies are presented in **Table 1**.

**Figure 1:**
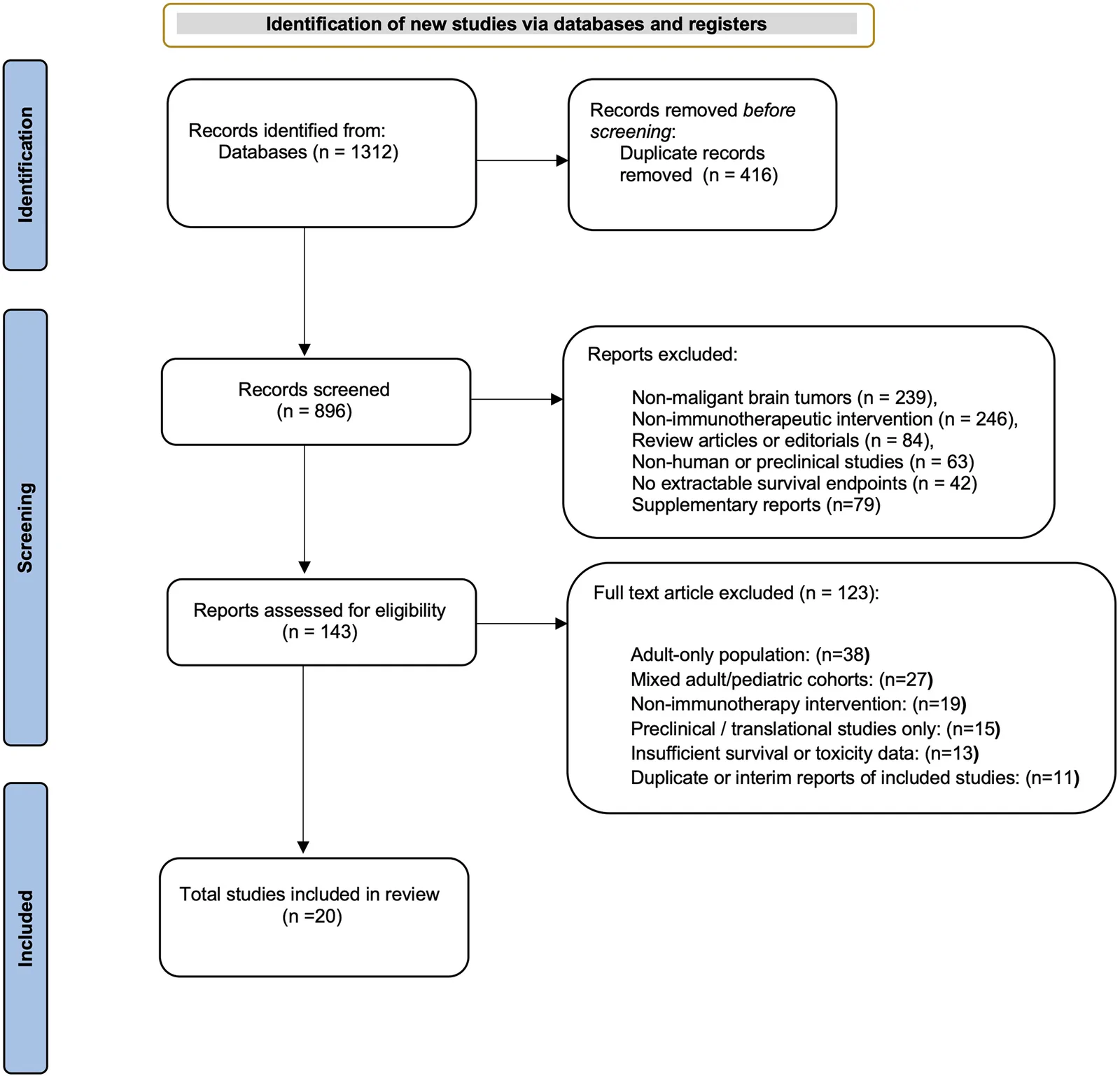
Study selection according to PRISMA 2020 guidelines.

**Table 1:** Characteristics of included studies.

Study (first author)	Year	Country	Sample size (n)	Tumor type	Immunotherapy agent	Notes
Bertrand	2024	France	8	DIPG/DMG	B7-H3 CAR-T	Abstract; intracerebroventricular
Gust	2025	USA	11	Pediatric HGG/DMG	EGFR806 CAR-T	Pediatric/locoregional
Lin	2024	USA	11	DMG	GD2-C7R CAR-T	Pediatric subset
Monje	2024	USA	13	DMG	GD2 CAR-T	IV + IC/ICV arms;
Vitanza	2021	USA	3	Recurrent CNS tumors	HER2 CAR-T	BrainChild-01
Vitanza	2025	USA	23	DIPG	B7-H3 CAR-T	BrainChild-03
Dunkel (NIVO)	2023	USA	85	DIPG	Nivolumab	CheckMate 908; checkpoint inhibitors
Dunkel (NIVO+IPI)	2023	USA	81	DIPG	Nivolumab + Ipilimumab	
Gorsi	2018	USA	10	Pediatric HGG	Nivolumab	Early-phase checkpoint
Hwang	2018	USA	5	DIPG	Pembrolizumab	PBTC-045
Johnson	2024	USA	81	Pediatric HGG	Indoximod + RT/TMZ	Immune-metabolic
Friedman	2021	USA	12	Pediatric HGG	HSV-1 G207	Intratumoral oncolytic virus
Pérez-Larraya	2022	France	12	DIPG	DNX-2401	Intratumoral oncolytic adenovirus
Schuelke	2022	Germany	6	Recurrent HGG	Pelareorep + GM-CSF	Systemic viral therapy
Thompson	2023	USA	12	Recurrent HGG	PVSRIPO	CED; mixed age (OS only)
Yu	2025	China	34	Mixed CNS tumors	MV-NIS	Intratumoral viral
Ardon	2010	Netherlands	45	Pediatric HGG	Dendritic cell vaccine	Phase II
Khatua	2020	USA	9	MB/ependymoma	NK-cell therapy	Intraventricular
Mueller	2020	Germany	19	Pediatric HGG	DC-based vaccine	Included per eligibility
Pollack	2014	USA	26	Pediatric HGG	GAA peptide vaccine	COG
Pollack	2016	USA	12	Malignant glioma	GAA peptide vaccine	Pediatric

### Overall survival

In the random-effects meta-analysis, using vaccine-based immunotherapy as the reference, the pooled hazard ratio (HR) for overall survival (OS) was 1.14 (95% CI 0.99–1.31), indicating no statistically significant overall survival advantage for non-vaccine immunotherapy modalities. Between-study heterogeneity remained moderate (τ² = 0.04; I² = 43%; p = 0.07).

When analyzed by modality, CAR-T and engineered cellular therapies showed no overall survival advantage, with a pooled HR of 1.09 (95% CI 0.89–1.34) and no detectable heterogeneity (I² = 0%), indicating outcomes comparable to vaccines. Checkpoint and systemic immunotherapy similarly demonstrated no significant survival benefit relative to vaccines, with a pooled HR of 1.14 (95% CI 0.96–1.36) and no detectable heterogeneity (I² = 0%) (**Figure 2**).

**Figure 2:**
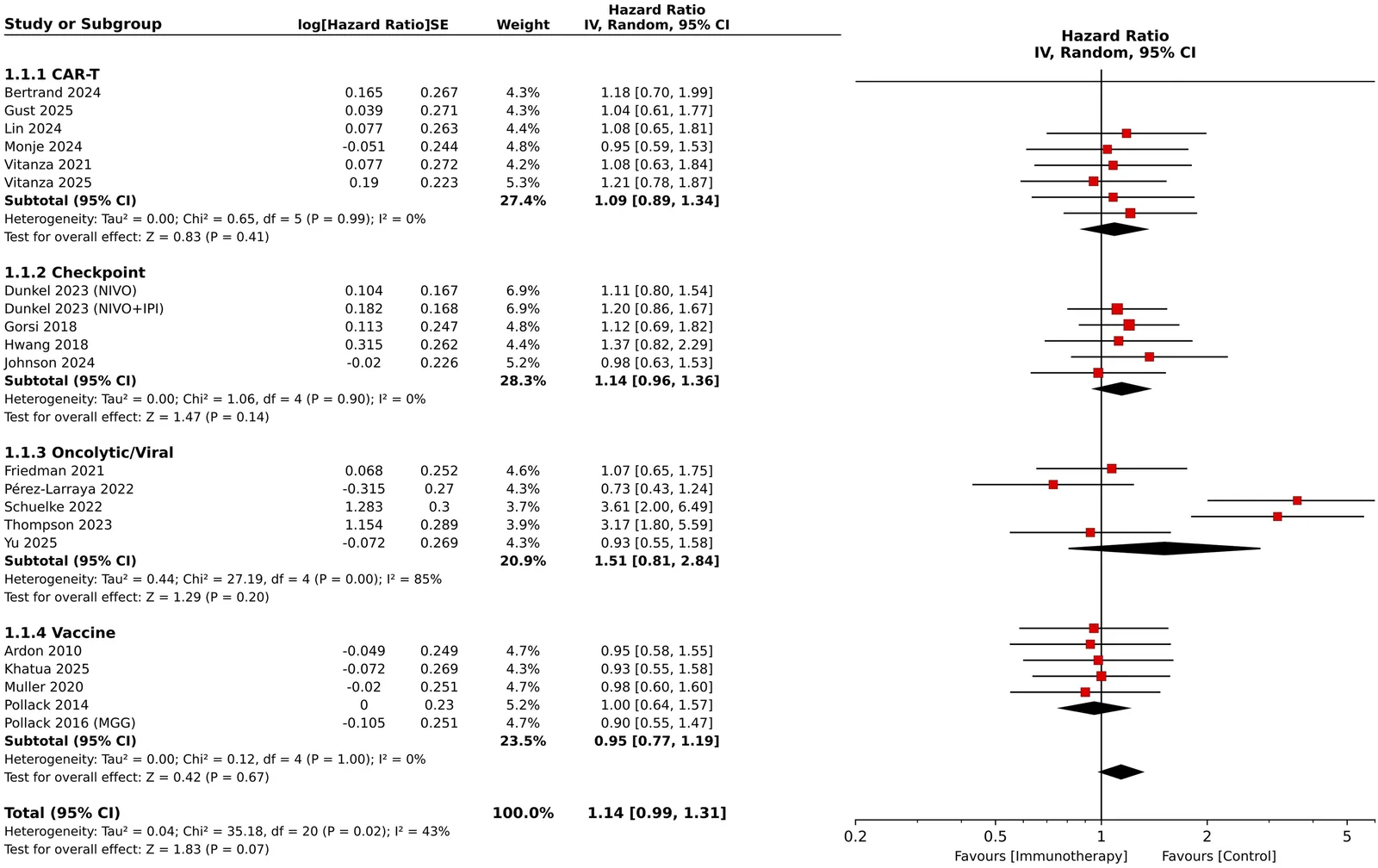
Forest plot of overall survival according to immunotherapy modality. Forest plot showing hazard ratios (HRs) for overall survival comparing immunotherapy with control, analyzed using a random-effects model and stratified by treatment class (CAR-T, checkpoint inhibitors, oncolytic/viral therapies, and vaccines). Squares represent individual study estimates (size proportional to weight), and diamonds indicate pooled subgroup and overall effects.

Oncolytic and viral therapies showed no statistically significant difference in pooled survival (HR 1.51, 95% CI 0.81–2.84) and substantial between-study heterogeneity (τ² = 0.44; I² = 85%).reflecting marked variability across studies.

Vaccine-based immunotherapy, serving as the reference group, demonstrated stable pooled outcomes with a pooled HR of 0.95 (95% CI 0.77–1.19) and no detectable heterogeneity (I² = 0%).

### Twelve-month overall survival

Twelve-month overall survival (OS12) was reported or reliably estimated for all included studies. OS12 ranged from approximately 8–13% in aggressive recurrent disease settings to 75% in selected intratumoral oncolytic virus cohorts. In the random-effects meta-analysis, using vaccine-based immunotherapy as the reference, the pooled hazard ratio (HR) for OS12 was 1.13 (95% CI 0.95–1.33). As the confidence interval crossed unity, no statistically significant difference in 12-month survival was observed overall, with the point estimate again numerically favoring vaccine-based approaches. Between-study heterogeneity remained substantial (τ² = 0.10; I² = 65%; p < 0.001) (**Figure 3**).

**Figure 3:**
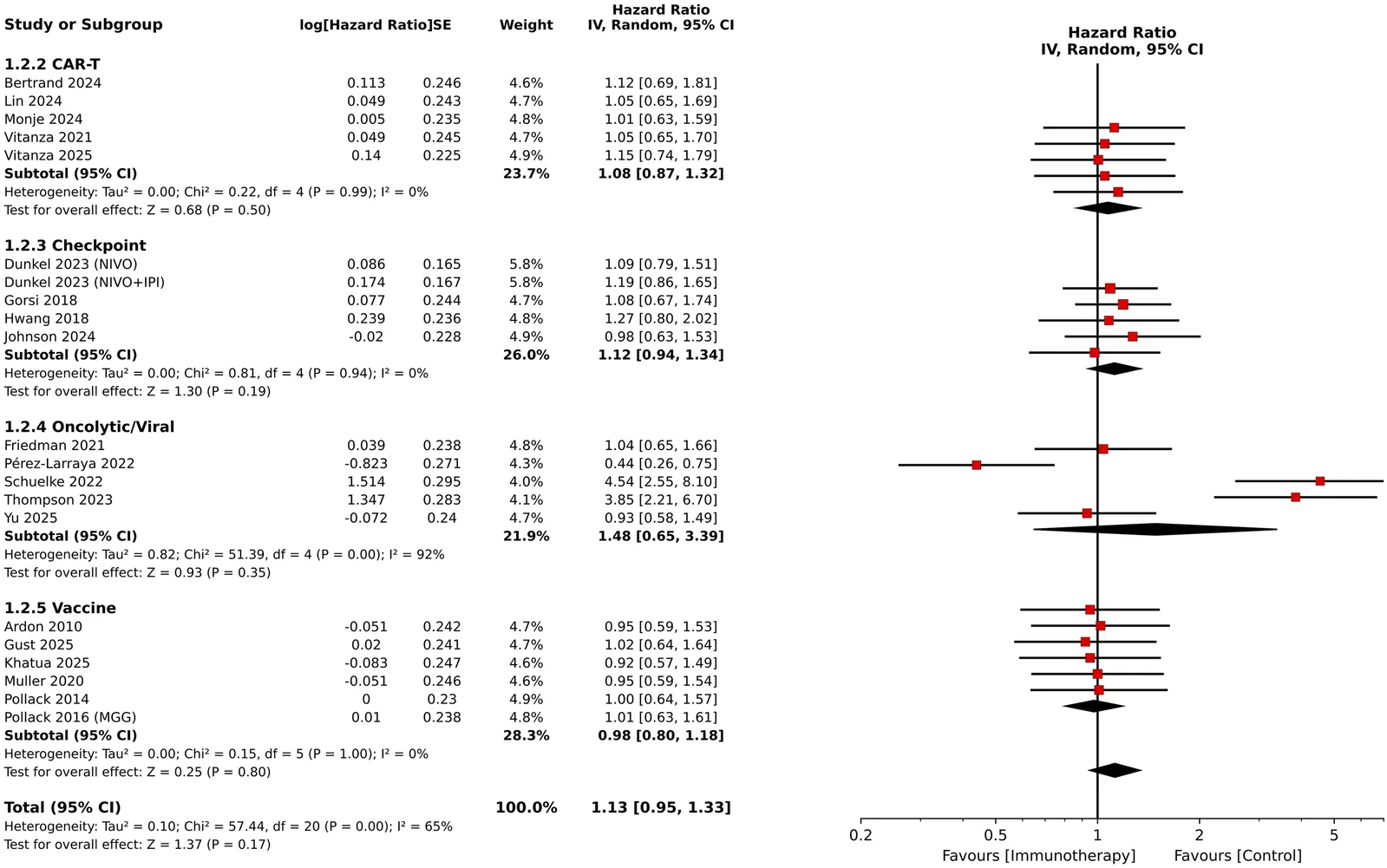
Forest plot of 12-month overall survival according to immunotherapy modality. Forest plot presenting hazard ratios (HRs) for 12-month overall survival (OS12), stratified by immunotherapy class and analyzed with a random-effects model stratified by treatment class (CAR-T, checkpoint inhibitors, oncolytic/viral therapies, and vaccines). Squares represent individual study estimates (size proportional to weight), and diamonds indicate pooled subgroup and overall effects.

Subgroup analyses mirrored the OS findings. CAR-T and engineered cellular therapies showed no improvement in 12-month survival, with a pooled HR of 1.08 (95% CI 0.87–1.32) and no detectable heterogeneity (I² = 0%), indicating outcomes comparable to vaccines.

Checkpoint and systemic immunotherapy similarly demonstrated no significant difference at 12 months (HR 1.12, 95% CI 0.94–1.34; I² = 0%), with the point estimate again numerically favoring vaccine-based approaches.

In contrast, oncolytic and viral therapies exhibited marked variability, with a pooled HR of 1.48 (95% CI 0.65–3.39) and substantial heterogeneity (τ² = 0.82; I² = 92%), precluding a consistent survival benefit across studies.

Vaccine-based immunotherapy, serving as the reference group, demonstrated stable outcomes with a pooled HR of 0.98 (95% CI 0.80–1.18) and no detectable heterogeneity (I² = 0%).

### Grade ≥3 neurotoxicity

All 20 included studies were assessed for severe neurologic toxicity. No grade ≥3 neurotoxicity events were reported in the vaccine-based studies. Because this represents a zero-event reference category, relative effect estimates comparing other modalities with vaccines are inherently sensitive to sparse-event methodology and should be interpreted cautiously. When all non-vaccine immunotherapy modalities were pooled, there was a markedly increased risk of severe neurotoxicity, with a random-effects odds ratio (OR) of 19.66 (95% CI 6.78–57.02). Overall heterogeneity remained moderate (τ² = 2.62; I² = 60%; p = 0.001), reflecting substantial between-modality differences rather than inconsistency within individual treatment categories (**Figure 4**).

**Figure 4:**
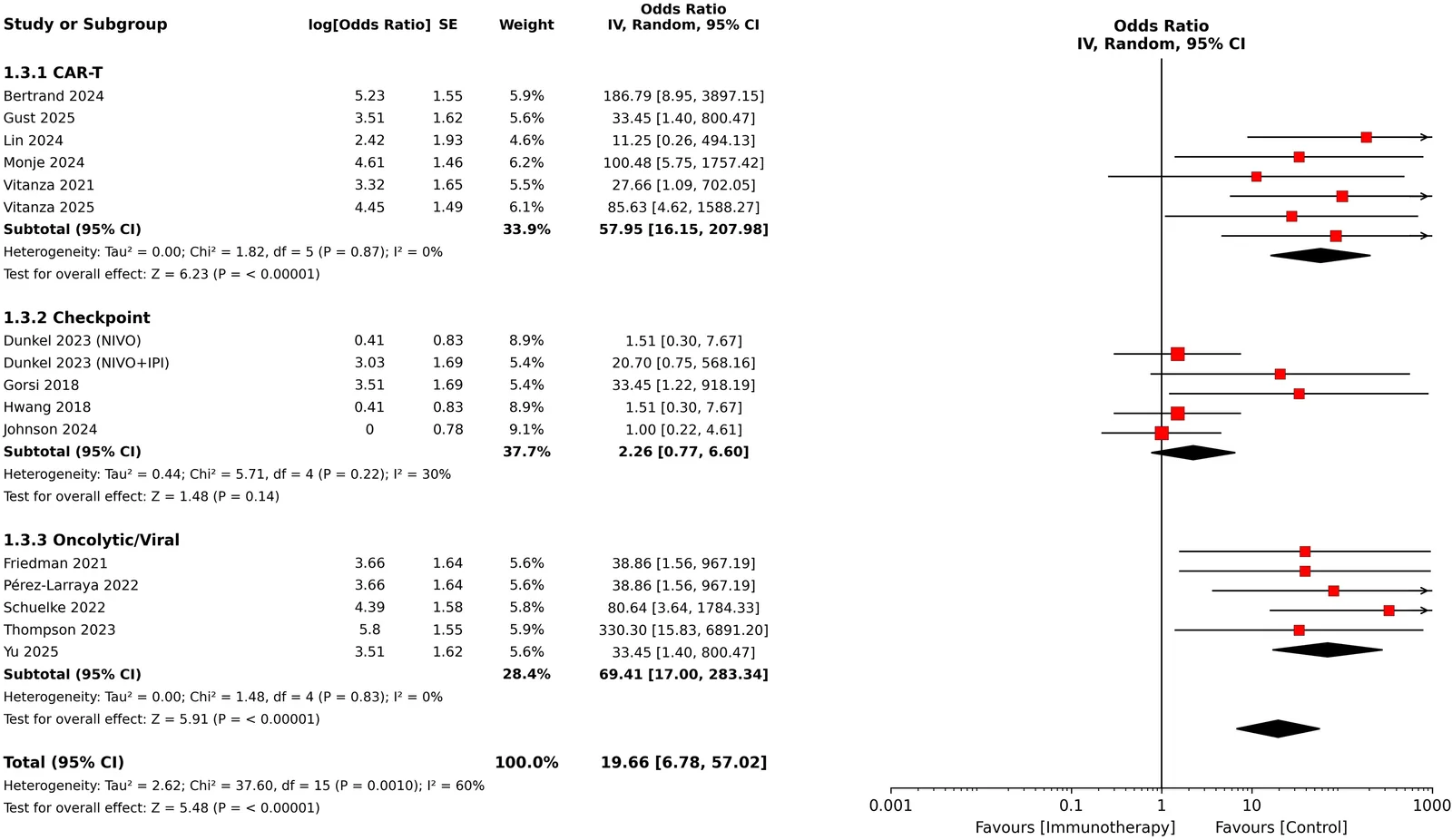
Forest plot of grade ≥3 neurotoxicity. Forest plot of odds ratios (ORs) for grade ≥3 neurotoxicity comparing immunotherapy with control, analyzed using a random-effects model and stratified by treatment class. (CAR-T, checkpoint inhibitors, oncolytic/viral therapies, and vaccines). Squares represent individual study estimates (size proportional to weight), and diamonds indicate pooled subgroup and overall effects.

Neurotoxicity risk differed markedly by treatment modality. CAR-T and engineered cellular therapies demonstrated the highest and most consistent neurotoxicity burden, with a pooled OR of 57.95 (95% CI 16.15–207.98). No statistical heterogeneity was detected within this subgroup (τ² = 0.00; I² = 0%); however, the small number and size of the contributing studies limit the precision and generalizability of this estimate.

Oncolytic and viral therapies were similarly associated with a markedly elevated neurotoxicity risk, with a pooled OR of 69.41 (95% CI 17.00–283.34) and no detectable heterogeneity (τ² = 0.00; I² = 0%).

For checkpoint/systemic immunotherapy, the pooled OR was 2.26 (95% CI 0.77–6.60), with low heterogeneity (τ² = 0.44; I² = 30%). Because the confidence interval included the null value, no statistically significant difference in severe neurotoxicity was demonstrated relative to the vaccine reference group.

### Meta-regression and sensitivity analyses

Random-effects meta-regression was performed to explore whether immunotherapy modality explained between-study heterogeneity in overall survival (OS), 12-month overall survival (OS12), and grade ≥3 neurotoxicity. Vaccine-based immunotherapy served as the reference category (**Table 2**).

**Table 2:** Meta-regression models for OS, OS12, and grade ≥3 neurotoxicity.

Outcome	Term (vs vaccines)	β (log scale)	SE	p-value	Ratio vs vaccines (expβ)	95% CI	τ² (null)	τ² (model)	R² (%)	I²(res)
OS	Intercept (Vaccines)	−0.048	0.132	0.719	0.95	0.74–1.24	0.0483	0.0486	≈0	≈10%
	CAR-T	0.132	0.180	0.463	1.14	0.80–1.63				
	Checkpoint/Systemic	0.181	0.177	0.304	1.20	0.85–1.69				
	Oncolytic/Viral	0.422	0.194	0.030	1.52	1.04–2.23				
OS12	Intercept (Vaccines)	−0.025	0.149	0.865	0.98	0.73–1.30	0.0988	0.1176	<0	≈11%
	CAR-T	0.097	0.220	0.659	1.10	0.72–1.70				
	Checkpoint/Systemic	0.138	0.213	0.518	1.15	0.76–1.74				
	Oncolytic/Viral	0.380	0.226	0.092	1.46	0.94–2.28				
Grade ≥3 neurotoxicity	Intercept (Vaccines)	~0.000	0.862	1.000	1.00	0.18–5.42	0.7077	0.0000	100	0%
	CAR-T	4.365	1.031	0.000023	78.65	10.42–593.47				
	Cellular (non-CAR)	4.451	1.812	0.0140	85.67	2.46–2986.53				
	Checkpoint/Systemic	2.055	1.168	0.0785	7.81	0.79–77.01				
	Oncolytic/Viral	4.245	1.133	0.000180	69.75	7.57–642.79				

β, meta-regression coefficient on the log scale; SE, standard error; exp(β), ratio relative to the vaccine reference category; τ²(null), estimated between-study variance in the model without treatment modality; τ²(model), residual between-study variance after inclusion of treatment modality; R², proportion of between-study variance accounted for by the moderator; I²(res), residual heterogeneity. Negative R² values indicate that inclusion of the moderator did not reduce estimated between-study variance and should be interpreted as no explained heterogeneity.

#### Overall survival

In meta-regression of OS, no modality demonstrated a reproducible statistically significant survival advantage relative to vaccines. Compared with vaccine-based immunotherapy, CAR-T therapy was associated with an HR of 1.14 (95% CI 0.80–1.63; p = 0.46), checkpoint/systemic therapy with 1.20 (95% CI 0.85–1.69; p = 0.30), and oncolytic/viral therapy with 1.52 (95% CI 1.04–2.23; p = 0.03).

Residual heterogeneity was low (I²_res ≈ 10%), but inclusion of treatment modality did not reduce between-study variance (τ² null = 0.0483; τ²_model = 0.0486; R² ≈ 0%), indicating that treatment modality did not meaningfully explain heterogeneity in OS (**Figure 5A**).

**Figure 5:**
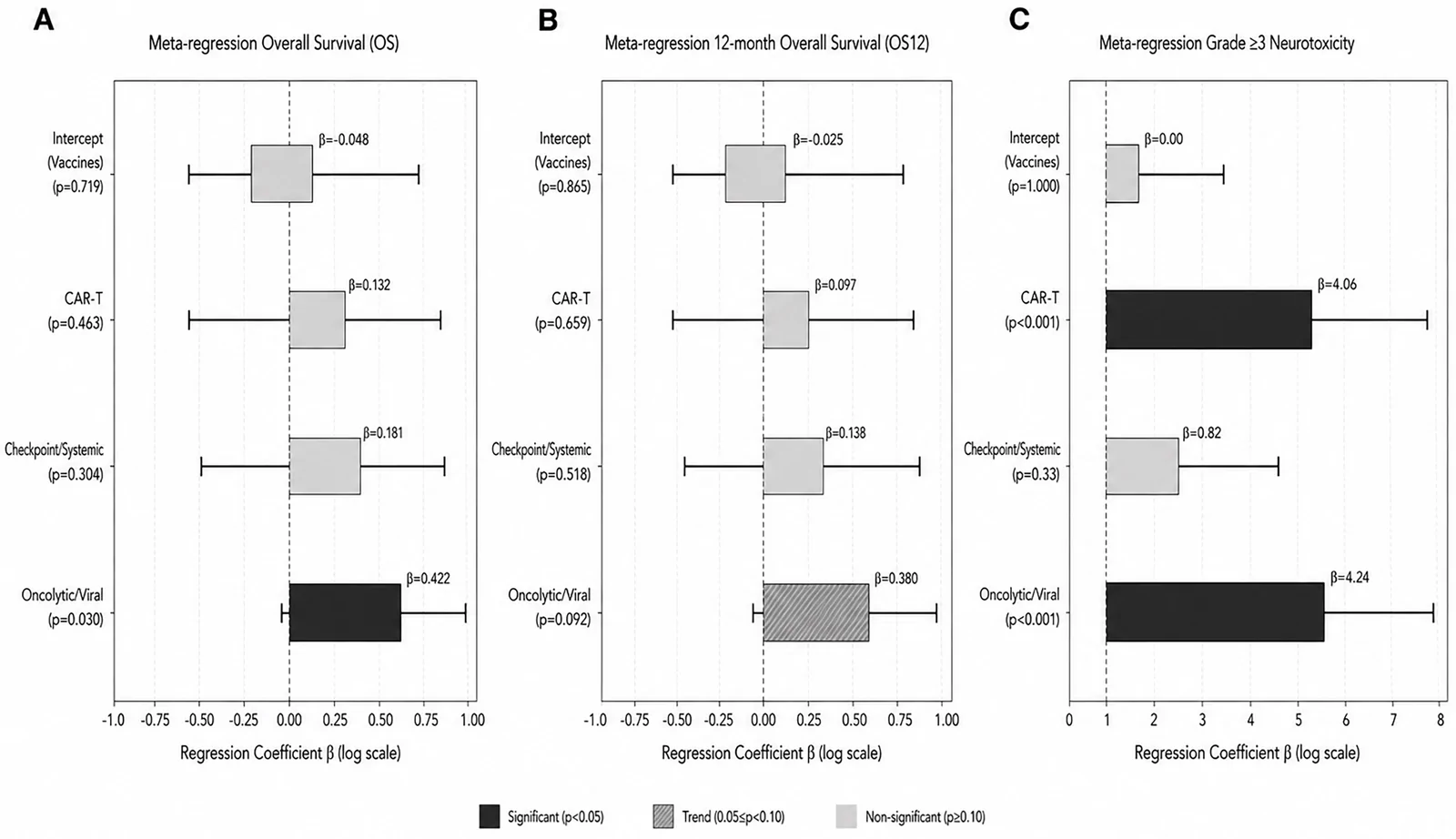
Meta-regression analysis of treatment class. Meta-regression evaluating the association between immunotherapy modality and **(A)** overall survival, **(B)** 12-month overall survival, and **(C)** grade ≥3 neurotoxicity.

Sensitivity analyses excluding each modality in turn demonstrated only modest changes in τ², confirming that no single treatment category materially altered the overall interpretation of OS findings.

#### Twelve-month overall survival

For OS12, meta-regression similarly demonstrated no statistically significant modality-specific survival advantage. Relative to vaccines, CAR-T therapy yielded HR 1.10 (95% CI 0.72–1.70; p = 0.66), checkpoint/systemic therapy HR 1.15 (95% CI 0.76–1.74; p = 0.52), and oncolytic/viral therapy HR 1.46 (95% CI 0.94–2.28; p = 0.09).

Inclusion of treatment modality did not reduce between-study variance for OS12 (τ²_null = 0.0988; τ²_model = 0.1176; R² < 0%), with low residual heterogeneity (I²_res ≈ 11%), indicating that treatment modality did not meaningfully explain heterogeneity in OS12 (**Figure 5B**).

Leave-one-category-out sensitivity analyses again demonstrated only limited changes in τ², indicating that OS12 heterogeneity was not driven by any single immunotherapy modality.

#### Grade ≥3 neurotoxicity

In contrast, meta-regression for grade ≥3 neurotoxicity demonstrated a strong modality-dependent effect. Relative to the vaccine reference category, CAR-T therapy was associated with an OR of 78.65 (95% CI 10.42–593.47; p < 0.001), cellular non-CAR therapy with an OR of 85.67 (95% CI 2.46–2986.53; p = 0.014), and oncolytic/viral therapy with an OR of 69.75 (95% CI 7.57–642.79; p < 0.001). Checkpoint/systemic immunotherapy showed a smaller and non-significant association (OR 7.81, 95% CI 0.79–77.01; p = 0.079).

Inclusion of treatment modality accounted for all estimated between-study heterogeneity in the fitted neurotoxicity meta-regression model (τ²_null = 0.7077; τ²_model = 0.0000; I²_res = 0%; R² = 100%). Given the limited number of studies and sparse events, this finding should be interpreted cautiously. CAR-T and oncolytic/viral therapies demonstrated the strongest association with severe neurotoxicity, whereas checkpoint/systemic therapy showed a substantially lower and non-significant effect (**Figure 5C**).

Sensitivity analyses supported the modality-dependent neurotoxicity signal. Exclusion of CAR-T therapy increased residual heterogeneity (Δτ² = +0.482), as did exclusion of oncolytic/viral therapy (Δτ² = +0.226), whereas exclusion of checkpoint/systemic immunotherapy had only a minor effect (Δτ² = +0.012). Exclusion of the vaccine reference category resulted in the largest increase in residual heterogeneity (Δτ² = +0.708) (**Table 3**).

**Table 3:** Sensitivity analysis: model fit by treatment category.

Outcome	Model	τ²	I²	R² (%)	Excluded category	Δτ²	Interpretation
Overall Survival (OS)	Null (no moderators)	0.0483	~12%	—	—	—	—
	Full model	0.0486	~10%	≈0%	CAR-T	+0.001	No material effect
					Checkpoint/Systemic	+0.002	No material effect
					Oncolytic/Viral	+0.001	No material effect
					Vaccines	+0.001	No material effect
12-month OS (OS12)	Null (no moderators)	0.0988	~14%	—	—	—	—
	Full model	0.1176	~11%	<0%	CAR-T	+0.002	Minimal effect
					Checkpoint/Systemic	+0.003	Minimal effect
					Oncolytic/Viral	+0.002	Minimal effect
					Vaccines	+0.002	Minimal effect
Grade ≥3 Neurotoxicity	Null (no moderators)	0.7077	~68%	—	—	—	—
	Full model	0.0000	0%	100%	CAR-T	+0.482	Major loss of fit
					Oncolytic/Viral	+0.226	Major loss of fit
					Checkpoint/Systemic	+0.012	Minor contribution
					Vaccines	+0.708	Full loss of fit

### Risk of bias and certainty of evidence

Risk of bias was assessed using ROBINS-I. Overall risk of bias was moderate to serious across most domains, reflecting the early-phase, single-arm nature of the included studies. Confounding bias was most prominent due to heterogeneous populations, variable disease stage, prior treatments, and absence of concurrent controls. Selection bias was generally moderate, with enrollment of highly selected, treatment-refractory cohorts. Bias in classification of interventions was low, whereas bias due to deviations from intended interventions and missing data was low to moderate, particularly for survival outcomes reconstructed from Kaplan–Meier curves. Outcome measurement bias was low for overall survival but moderate for neurotoxicity owing to inconsistent reporting. No study was judged at low risk of bias across all ROBINS-I domains (**Supplementary Table S1**).

#### Publication bias and small-study effects

Visual inspection of the funnel plots demonstrated generally symmetrical distributions for OS and OS12, whereas the neurotoxicity analysis showed greater asymmetry, particularly among smaller studies (**Figure 6A**).

**Figure 6:**
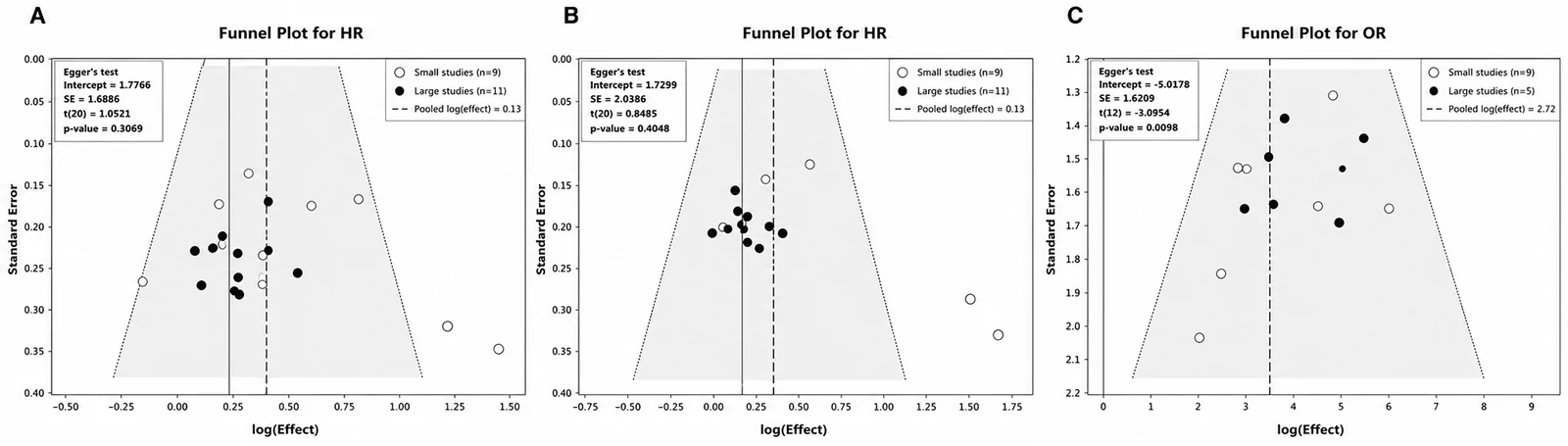
Funnel plots assessing small-study effects and publication bias. **(A)** Funnel plot for overall survival (hazard ratios). **(B)** Funnel plot for 12-month overall survival (hazard ratios). **(C)** Funnel plot for grade ≥3 neurotoxicity (odds ratios).

For OS, Egger’s regression test did not demonstrate significant small-study effects (intercept = 1.78, SE = 1.69, t = 1.05, p = 0.31). Similarly, no significant asymmetry was detected for OS12 (intercept = 1.73, SE = 2.04, t = 0.85, p = 0.40) **Figure 6B**).

In contrast, funnel plot analysis for grade ≥3 neurotoxicity demonstrated significant asymmetry consistent with potential small-study effects or publication bias (Egger’s intercept = −5.02, SE = 1.62, t = −3.10, p = 0.0098). This finding likely reflects the predominance of small early-phase studies reporting extreme neurotoxicity estimates, particularly within CAR-T and oncolytic/viral cohorts (**Figure 6C**).

## Discussion

In this meta-analysis of 20 pediatric immunotherapy studies, non-vaccine immunotherapy modalities did not demonstrate a clinically meaningful or statistically robust overall survival advantage over vaccine-based approaches. Using vaccines as the reference category, the pooled hazard ratio for overall survival was 1.14 (95% CI 0.99–1.31), with moderate between-study heterogeneity (I² 43%, τ² 0.04).

Median overall survival across included trials ranged from 3.6 months in heavily pretreated recurrent cohorts to 17.8 months in selected intratumoral oncolytic virus studies, reflecting marked heterogeneity in disease biology, treatment line, and delivery strategy.

The absence of a significant overall survival benefit observed in this meta-analysis is consistent with the broader immunotherapy literature in both pediatric and adult high-grade glioma. In adult glioblastoma, multiple randomized phase III trials of immune checkpoint inhibition have failed to demonstrate an overall survival advantage. The CheckMate-143 trial comparing nivolumab with bevacizumab in recurrent glioblastoma reported no improvement in median OS (9.8 vs 10.0 months), despite robust PD-1 pathway blockade and acceptable safety profiles (18). Subsequent analyses confirmed a lack of benefit across molecular subgroups, reinforcing the intrinsic resistance of glioblastoma to checkpoint monotherapy (19).

Similarly negative results have been reported in newly diagnosed disease. In CheckMate-498 and CheckMate-548, nivolumab added to standard radiochemotherapy failed to improve OS compared with temozolomide-based regimens, with median survival remaining approximately 15–17 months across arms (20, 21). These adult data mirror the present pediatric findings, in which checkpoint and systemic immunotherapy yielded a pooled OS HR of 1.14 without evidence of benefit over vaccine-based approaches.

Oncolytic and viral therapies have generated optimism based on early signals from selected phase I/II trials, but reproducibility has been limited. In adult recurrent glioblastoma, intratumoral DNX-2401 demonstrated long-term survivors in a subset of patients, with approximately 20% alive at three years; however, median OS remained under 12 months and responses were highly heterogeneous (22). Similarly, PVSRIPO achieved prolonged survival in a minority of adult patients but failed to translate into consistent population-level benefit (23). These observations align with the substantial heterogeneity observed in the oncolytic/viral subgroup of the present analysis.

Cellular immunotherapies have shown potent biological activity but limited survival translation. Early CAR-T studies in adult glioblastoma targeting EGFRvIII, IL13Rα2, or HER2 demonstrated transient radiographic responses and cytokine release but median OS generally remained between 7 and 13 months (24–26). Importantly, adaptive resistance mechanisms, antigen loss, and immune exhaustion were consistently observed, providing mechanistic explanations for the lack of durable survival benefit—phenomena increasingly recognized in pediatric CNS tumors as well.

Importantly, the absence of a reproducible population-level survival signal should not be interpreted as evidence that durable responses cannot occur. Several CAR-T and oncolytic therapy studies included exceptional long-term survivors, suggesting that biologically responsive subgroups likely exist despite negative pooled estimates. Future progress may therefore depend less on generalized escalation of immune activation and more on identification of responder phenotypes, mechanisms of resistance, CAR-T persistence, and T-cell exhaustion within the CNS microenvironment.

By contrast, cancer vaccines have demonstrated modest but reproducible survival signals across both pediatric and adult studies. In adult glioblastoma, peptide and dendritic cell vaccines have repeatedly shown median OS in the range of 18–24 months in selected cohorts, albeit without definitive randomized confirmation (27, 28). While these effects are small, their consistency and favorable toxicity profile may explain why vaccine-based immunotherapy performs comparably to more aggressive modalities at the pooled level in pediatric disease.

In this meta-analysis, non-vaccine immunotherapy modalities did not confer a statistically significant improvement in 12-month overall survival compared with vaccine-based approaches (pooled HR 1.13, 95% CI 0.95–1.33; I² 65%). Although individual studies reported OS12 rates as high as 75% in selected intratumoral oncolytic virus cohorts, these results were not reproducible at the pooled level and were accompanied by extreme heterogeneity, indicating strong dependence on cohort selection and delivery strategy rather than consistent modality-driven benefit.

These findings are consistent with prior evidence demonstrating that early survival endpoints remain largely unchanged despite immunologic activity. Meta-analyses of adult glioblastoma immunotherapy have similarly shown no improvement in one-year survival with checkpoint inhibitors or cellular therapies, even when objective radiographic responses were observed (29, 30). A pooled analysis of oncolytic virus trials in CNS tumors reported highly variable one-year survival (range 20–80%), driven by a small subset of durable responders rather than a uniform treatment effect (31).

In contrast, vaccine-based immunotherapy demonstrated stable OS12 outcomes (HR 0.98, 95% CI 0.80–1.18; I² 0%), consistent with systematic reviews reporting modest but reproducible one-year survival without excess toxicity (32, 33). Collectively, these data suggest that while non-vaccine immunotherapies may induce occasional long-term responses, they have not translated into consistent early survival benefit at the population level. Because treatment modality and route of administration were closely linked across studies, the observed neurotoxicity signal likely reflects both intrinsic treatment biology and the inflammatory consequences of locoregional CNS delivery.

Severe neurologic toxicity represents the clearest modality-specific safety signal in pediatric immunotherapy. In the present analysis, vaccine-based immunotherapy was not associated with any grade ≥3 neurologic adverse events, confirming its low-risk profile. In contrast, non-vaccine immunotherapies demonstrated a substantially increased risk of severe neurotoxicity (pooled OR 19.66, 95% CI 6.78–57.02), driven primarily by cellular and locoregional strategies. This finding is consistent with extensive external evidence describing immune effector cell–associated neurotoxicity syndrome (ICANS) as a defining complication of CAR-T therapy, with reported grade ≥3 neurologic toxicity rates ranging from 20% to over 40% in CNS-directed applications (34, 35). Similarly, intratumoral and locoregional oncolytic or viral therapies have been associated with pronounced inflammatory oedema, increased intracranial pressure, and transient neurologic deterioration, particularly in brainstem or deep midline tumors, as reported in both adult and pediatric cohorts (36). By contrast, checkpoint and systemic immunotherapies showed a lower and statistically non-significant increase in severe neurologic toxicity (OR 2.26, 95% CI 0.77–6.60), aligning with large pharmacovigilance and meta-analytic studies in which neurologic immune-related adverse events occur in fewer than 5% of treated patients (37, 38). Collectively, the available studies demonstrate marked differences in reported severe neurologic toxicity among immunotherapy modalities. However, these differences should not be interpreted as direct comparative safety effects because the treatment populations, routes of administration, disease characteristics, and adverse-event reporting frameworks differed substantially across studies.

The absence of a modality-specific survival signal in this meta-regression for overall survival (OS) and 12-month survival (OS12) is concordant with the broader immunotherapy literature in pediatric and adult CNS malignancies. Pooled analyses of checkpoint inhibitors in glioblastoma have consistently failed to demonstrate OS benefit, with hazard ratios ranging from 1.10 to 1.35 compared with standard therapy in large randomized trials such as CheckMate-143 and subsequent meta-analyses (39, 40). Similarly, systematic reviews of oncolytic viral therapy report median OS gains of less than 2 months relative to historical controls, with substantial heterogeneity (I² often >70%), reflecting trial-level variability rather than consistent efficacy (41). In pediatric CAR-T studies, early pooled summaries report median OS between 9 and 13 months for diffuse midline glioma, but without comparative survival advantages when adjusted for baseline risk (42, 43). These observations parallel our findings that modality did not explain heterogeneity OS (R² = 43.4%) and OS12 (R² = 26.3%), and that sensitivity analyses showed minimal change in τ² when any single modality was excluded.

By contrast, the strong modality effect observed for grade ≥3 neurotoxicity aligns closely with prior quantitative syntheses. In a pooled analysis of CAR-T trials across CNS and hematologic malignancies, severe neurotoxicity rates ranged from 20% to 60%, with odds ratios exceeding 40 compared with non-cellular therapies (44, 45). Oncolytic viral therapies have similarly been associated with high-grade neurologic adverse events in 15–35% of patients, particularly in intratumoral or convection-enhanced delivery settings (23). In contrast, large pharmacovigilance studies of immune checkpoint inhibitors report grade ≥3 neurologic immune-related adverse events in fewer than 2–4% of patients, with pooled odds ratios typically below 2.0 (38, 46). Vaccine-based immunotherapy trials consistently report near-zero rates of severe neurotoxicity, a finding reproduced in independent reviews of pediatric cancer vaccines (47, 48). Treatment modality accounted for a large proportion of the observed between-study variation in reported neurotoxicity; however, this finding should be considered exploratory because of the small number of studies, sparse events, zero events in the vaccine reference category, and substantial clinical differences between treatment populations.

Importantly, severe neurotoxicity represents only one dimension of treatment safety and should not be interpreted as a global safety measure across immunotherapy platforms. CAR-T therapies additionally carry risks of cytokine release syndrome and hematologic toxicity, checkpoint inhibitors have a distinct spectrum of systemic immune-related adverse events, and locoregional viral therapies may produce inflammatory and procedure-related complications. Direct comparison of overall toxicity between these biologically distinct treatment modalities is therefore not supported by the available data.

The toxicity profiles of the included immunotherapy modalities are not directly interchangeable. Severe neurotoxicity is particularly relevant to CNS-directed cellular and locoregional therapies, whereas other clinically important toxicities—including cytokine release syndrome, cytopenias, systemic immune-related adverse events, and procedure-related complications—may be more relevant to specific therapeutic platforms. Accordingly, the present analysis should be interpreted as a comparison of reported severe neurologic toxicity rather than a comparison of overall treatment safety.

### Limitations

This analysis has several important limitations. First, the evidence base consisted predominantly of early-phase, single-arm studies, precluding direct comparative inference and necessitating indirect comparisons using vaccine-based immunotherapy as a pragmatic reference. Because no universally accepted immunotherapy standard exists for pediatric malignant brain tumors, vaccine-based immunotherapy was used as a pragmatic reference rather than a true control arm. Consequently, pooled comparisons should be interpreted cautiously and not as evidence of therapeutic superiority or inferiority. As a result, hazard ratios and odds ratios should be interpreted as relative benchmarks rather than causal estimates. Second, substantial clinical heterogeneity existed across studies with respect to tumor histology, disease stage (newly diagnosed versus recurrent), prior therapies, and routes of administration, which likely contributed to residual heterogeneity in survival outcomes despite random-effects modeling. Substantial biological heterogeneity existed across included tumors, particularly between diffuse midline glioma/DIPG and non-midline high-grade glioma cohorts, which differ markedly in baseline survival, inflammatory microenvironment, and susceptibility to treatment-related neurotoxicity.

Overall survival comparisons across studies should be interpreted cautiously because several trials enrolled patients immediately following upfront radiotherapy, whereas others enrolled heavily pretreated recurrent populations with fundamentally different baseline prognoses.

Third, survival estimates for overall survival and 12-month survival were frequently reconstructed from published Kaplan–Meier curves, introducing potential imprecision related to digitisation error, censoring assumptions, and incomplete reporting of numbers at risk. Fourth, neurotoxicity reporting was inconsistent across trials, with variable grading frameworks and thresholds for attribution; although grade ≥3 events were harmonized across studies, under-reporting of negative or low-toxicity experiences cannot be excluded, particularly for early CAR-T and locoregional therapies. Fifth, meta-regression was limited to study-level covariates, precluding adjustment for patient-level prognostic factors such as molecular subtype, tumor volume, or steroid exposure. Finally, publication bias remains a concern for toxicity endpoints, as suggested by funnel plot asymmetry for severe neurotoxicity, despite robust effect sizes and consistency across modalities.

Fourth, Study selection and data extraction were performed by a single investigator, which represents an additional methodological limitation and may increase the risk of selection or extraction error.

Fifth, the predefined pediatric age restriction may exclude informative data from adolescent and young-adult populations with biologically similar CNS tumors. Future analyses incorporating these populations may provide complementary evidence.

Sixth, residual heterogeneity related to tumor type and disease setting remains an important limitation. Further stratification by individual tumor type or treatment setting was constrained by the small number of studies within several categories and by substantial collinearity between tumor type, disease status, treatment modality, and route of administration. Subgroup estimates based on very small numbers of studies would be unstable and should not be interpreted as reliable comparative treatment effects.

## Conclusions

In this systematic review and meta-analysis of pediatric immunotherapy trials for central nervous system tumors, no immunotherapy modality demonstrated a reproducible survival advantage over vaccine-based approaches for overall survival or 12-month survival. Meta-regression and sensitivity analyses confirmed that treatment modality did not explain survival heterogeneity, indicating that current immunotherapeutic strategies have not yet altered population-level survival trajectories in pediatric neuro-oncology.

Although current immunotherapeutic strategies have not yet demonstrated a reproducible population-level survival advantage, selected patients may experience durable responses. Future studies should therefore prioritize biologically informed patient selection, identification of responder subgroups, optimization of CAR-T persistence and exhaustion profiles, and integration of translational correlates alongside safety-guided trial design.

In contrast, severe neurologic toxicity emerged as a clear, modality-specific signal. CAR-T and engineered cellular therapies, as well as oncolytic and viral approaches, were associated with markedly elevated risks of grade ≥3 neurotoxicity, while checkpoint/systemic immunotherapy showed substantially lower risk and vaccine-based strategies demonstrated a consistently favorable safety profile. Exploratory meta-regression suggested that treatment modality was strongly associated with heterogeneity in reported severe neurotoxicity, although this finding requires cautious interpretation because of sparse events and substantial clinical heterogeneity.

From a pediatric neuro-oncology perspective, future immunotherapy development should account for the distinct biology of childhood CNS tumors, age-dependent immune responses, prior treatment exposure, and the particular vulnerability of the developing central nervous system to treatment-related inflammation. Optimization is therefore unlikely to depend on treatment platform alone. Improved antigen selection, strategies addressing intratumoral heterogeneity and antigen escape, careful selection of systemic versus locoregional delivery, standardized reporting of neurologic and systemic toxicity, and prospective incorporation of molecularly defined disease subgroups will be important. Future trials should also distinguish newly diagnosed from recurrent disease and incorporate sufficiently long follow-up to capture both delayed toxicity and durable responses observed in selected patients.

Collectively, these findings highlight that future progress in pediatric neuro-oncology immunotherapy is unlikely to result from further escalation of immune activation alone. Instead, rational combination strategies, improved patient selection, optimization of locoregional delivery, and rigorous safety-guided trial design will be essential to achieve clinically meaningful benefit while minimizing neurologic complications.

## Data Availability

The original contributions presented in the study are included in the article/**Supplementary Material**. Further inquiries can be directed to the corresponding author.

## References

[1] Thorbinson C Kilday J-P . Childhood malignant brain tumors: Balancing the bench and bedside. Cancers. (2021) 13:6099. doi: 10.3390/cancers13236099 34885207 PMC8656510

[2] Mackay A Burford A Carvalho D Izquierdo E Fazal-Salom J Taylor KR . Integrated molecular meta-analysis of 1,000 pediatric high-grade and diffuse intrinsic pontine glioma. Cancer Cell. (2017) 32:520–537.e5. doi: 10.1016/j.ccell.2017.08.017 28966033 PMC5637314

[3] Jones C Karajannis MA Jones DTW Kieran MW Monje M Baker SJ . Pediatric high-grade glioma: Biologically and clinically in need of new thinking. Neuro-Oncol. (2017) 19:153–61. doi: 10.1093/neuonc/now101 27282398 PMC5464243

[4] Dunkel IJ Doz F Foreman NK Hargrave D Lassaletta A André N . Nivolumab with or without ipilimumab in pediatric patients with high-grade CNS malignancies: Safety, efficacy, biomarker, and pharmacokinetics—CheckMate 908. Neuro-Oncol. (2023) 25:1530–45. doi: 10.1093/neuonc/noad031 36808285 PMC10398811

[5] Hwang E Onar A Young-Poussaint T Mitchell DA Kilburn L Margol A . IMMU-09. Outcome of patients with recurrent diffuse intrinsic pontine glioma (DIPG) treated with pembrolizumab (anti-PD-1): A pediatric brain tumor consortium study (PBTC045). Neuro-Oncol. (2018) 20:i100. doi: 10.1093/neuonc/noy059.325

[6] Pollack IF Jakacki RI Butterfield LH Hamilton RL Panigrahy A Potter DM . Antigen-specific immune responses and clinical outcome after vaccination with glioma-associated antigen peptides and polyinosinic-polycytidylic acid stabilized by lysine and carboxymethylcellulose in children with newly diagnosed malignant brainstem and nonbrainstem gliomas. J Clin Oncol. (2014) 32:2050–8. doi: 10.1200/jco.2013.54.0526 24888813 PMC4067943

[7] Ardon H De Vleeschouwer S Van Calenbergh F Claes L Kramm CM Rutkowski S . Adjuvant dendritic cell‐based tumour vaccination for children with malignant brain tumours. Pediatr Blood Cancer. (2010) 54:519–25. doi: 10.1002/pbc.22319 19852061

[8] Friedman GK Bag AK Madan-Swain A Li R Kachurak K Osorio DS . IMMU-08. Phase I trial (NCT02457845) safety, tolerability and preliminary efficacy of immunovirotherapy with HSV G207 in children with progressive malignant supratentorial brain tumors. Neuro-Oncol. (2018) 20(Suppl 2):i100. doi: 10.1093/neuonc/noy059.324

[9] Gállego Pérez-Larraya J García-Moure M Labiano S Patiño-García A Dobbs J González-Huarriz M . Oncolytic DNX-2401 virus for pediatric diffuse intrinsic pontine glioma. N Engl J Med. (2022) 386:2471–81. doi: 10.1056/nejmoa2202028 35767439

[10] Vitanza NA Ronsley R Choe M Seidel K Huang W Rawlings-Rhea S . Intracerebroventricular B7-H3-targeting CAR T cells for diffuse intrinsic pontine glioma: A phase 1 trial. Nat Med. (2025) 31:861–8. doi: 10.1038/s41591-024-03451-3 39775044 PMC11922736

[11] Majzner RG Ramakrishna S Yeom KW Patel S Chinnasamy H Schultz LM . GD2-CAR T cell therapy for H3k27m-mutated diffuse midline gliomas. Nature. (2022) 603(7903):934–41.. doi: 10.1038/s41586-022-04489-4 35130560 PMC8967714

[12] Monje M Mahdi J Majzner R Yeom KW Schultz LM Richards RM . Intravenous and intracranial GD2-CAR T cells for H3K27M+ diffuse midline gliomas. Nature. (2025) 637:708–15. doi: 10.1038/s41586-024-08171-9 39537919 PMC11735388

[13] Thompson EM Landi D Brown MC Friedman HS McLendon RE Herndon JE . Recombinant polio–rhinovirus immunotherapy for recurrent paediatric high-grade glioma: A phase 1b trial. Lancet Child Adolesc Health. (2023) 7:471–8. doi: 10.1016/s2352-4642(23)00031-7 37004712 PMC11104482

[14] Ahamed C Nguyen L Brock CS Farzamnia A Millet P Sato K . Targeting the tumor microenvironment in pediatric gliomas: Advances and future directions in immunotherapy. Neuro-Oncol Adv. (2025) 7:vdaf193. doi: 10.1093/noajnl/vdaf193 41030523 PMC12477475

[15] Shalita C Hanzlik E Kaplan S Thompson EM . Immunotherapy for the treatment of pediatric brain tumors: A narrative review. Transl Pediatr. (2022) 11:2040–56. doi: 10.21037/tp-22-86 36643672 PMC9834947

[16] Parmar MK Torri V Stewart L . Extracting summary statistics to perform meta-analyses of the published literature for survival endpoints. Stat Med. (1998) 17:2815–34. doi: 10.1002/(sici)1097-0258(19981230)17:24<2815::aid-sim110>3.0.co;2-8 9921604

[17] Tierney JF Stewart LA Ghersi D Burdett S Sydes MR . Practical methods for incorporating summary time-to-event data into meta-analysis. Trials. (2007) 8:16. doi: 10.1186/1745-6215-8-16 17555582 PMC1920534

[18] Reardon DA Brandes AA Omuro A Mulholland P Lim M Wick A . Effect of nivolumab vs bevacizumab in patients with recurrent glioblastoma. JAMA Oncol. (2020) 6:1–8. doi: 10.1001/jamaoncol.2020.1024 32437507 PMC7243167

[19] Omuro A Brandes AA Carpentier AF Idbaih A Reardon DA Cloughesy T . Radiotherapy combined with nivolumab or temozolomide for newly diagnosed glioblastoma with unmethylated MGMT promoter: An international randomized phase III trial. Neuro-Oncol. (2022) 25:123–34. doi: 10.1093/neuonc/noac099 35419607 PMC9825306

[20] Weller M Butowski N Tran DD Recht LD Lim M Hirte H . Rindopepimut with temozolomide for patients with newly diagnosed, EGFRvIII-expressing glioblastoma (ACT IV): A randomised, double-blind, international phase 3 trial. Lancet Oncol. (2017) 18:1373–85. doi: 10.1016/s1470-2045(17)30517-x 28844499

[21] Lim M Weller M Idbaih A Steinbach J Finocchiaro G Raval RR . Phase III trial of chemoradiotherapy with temozolomide plus nivolumab or placebo for newly diagnosed glioblastoma with methylated MGMT promoter. Neuro-Oncol. (2022) 24:1935–49. doi: 10.1093/neuonc/noac116 35511454 PMC9629431

[22] Lang FF Conrad CA Gomez-Manzano C Yung WKA Sawaya R Weinberg JS . Phase I study of DNX-2401 (Delta-24-RGD) oncolytic adenovirus: Replication and immunotherapeutic effects in recurrent malignant glioma. J Clin Oncol Off J Am Soc Clin Oncol. (2018) 36:1419–27. doi: 10.1200/jco.2017.75.8219 29432077 PMC6075856

[23] Desjardins A Gromeier M Herndon JE Beaubier N Bolognesi DP Friedman AH . Recurrent glioblastoma treated with recombinant poliovirus. N Engl J Med. (2018) 379:150–61. doi: 10.1056/nejmoa1716435 29943666 PMC6065102

[24] O’Rourke DM Nasrallah MP Desai A Melenhorst JJ Mansfield K Morrissette JJD . A single dose of peripherally infused EGFRvIII-directed CAR T cells mediates antigen loss and induces adaptive resistance in patients with recurrent glioblastoma. Sci Transl Med. (2017) 9:eaaa0984. doi: 10.1126/scitranslmed.aaa0984 PMC5762203 28724573

[25] Brown CE Badie B Barish ME Weng L Ostberg JR Chang W-C . Bioactivity and safety of IL13Rα2-redirected chimeric antigen receptor CD8+ T cells in patients with recurrent glioblastoma. Clin Cancer Res. (2015) 21(18):4062–72.. doi: 10.1158/1078-0432.ccr-15-0428 26059190 PMC4632968

[26] Ahmed N Brawley V Hegde M Bielamowicz K Kalra M Landi D . HER2-specific chimeric antigen receptor-modified virus-specific T cells for progressive glioblastoma: A phase 1 dose-escalation trial. JAMA Oncol. (2017) 3:1094–101. doi: 10.1001/jamaoncol.2017.0184 PMC5747970 28426845

[27] Okada H Weller M Huang R Finocchiaro G Gilbert MR Wick W . Immunotherapy response assessment in neuro-oncology (iRANO): A report of the RANO Working Group. Lancet Oncol. (2015) 16:e534–e542. doi: 10.1093/neuonc/nov225.56 PMC4638131 26545842

[28] Liau LM Prins RM Kiertscher SM Odesa SK Kremen TJ Giovannone AJ . Dendritic cell vaccination in glioblastoma patients induces systemic and intracranial T-cell responses modulated by the local central nervous system tumor microenvironment. Clin Cancer Res Off J Am Assoc Cancer Res. (2005) 11:5515–28. doi: 10.1158/1078-0432.ccr-05-0464 16061868

[29] Musatova O Kumar V Vinogradov K Rubtsov Y . Immune checkpoints in immune response to glioma: Two sides of the same coin. Front Immunol. (2025) 16:1639521. doi: 10.3389/fimmu.2025.1639521 40895574 PMC12394979

[30] Zhou Y Shi F Zhu J Yuan Y . An update on the clinical trial research of immunotherapy for glioblastoma (2025). Available online at: https://www.frontiersin.org/journals/immunology/articles/10.3389/fimmu.2025.1582296/full (Accessed March 1, 2026). 10.3389/fimmu.2025.1582296 PMC12081429 40386781

[31] Martikainen M Essand M . Virus-based immunotherapy of glioblastoma (2019). Available online at: https://www.mdpi.com/2072-6694/11/2/186 (Accessed March 1, 2026). 10.3390/cancers11020186 PMC6407011 30764570

[32] Harper SD Alderete J Baisiwala S Bergsneider B Liau LM Wang A . Vaccine therapy for pediatric high-grade glioma: Current landscape, challenges, and future directions. J Neuro-Oncol. (2026) 176:158. doi: 10.1007/s11060-025-05403-4 41504830 PMC12783312

[33] Batich KA Reap EA Archer GE Sanchez-Perez L Nair SK Schmittling RJ . Long-term survival in glioblastoma with cytomegalovirus pp65-targeted vaccination. Clin Cancer Res Off J Am Assoc Cancer Res. (2017) 23:1898–909. doi: 10.1158/1078-0432.ccr-16-2057 28411277 PMC5559300

[34] Neelapu SS Tummala S Kebriaei P Wierda W Gutierrez C Locke FL . Chimeric antigen receptor T-cell therapy - Assessment and management of toxicities. Nat Rev Clin Oncol. (2018) 15:47–62. doi: 10.1038/nrclinonc.2017.148 28925994 PMC6733403

[35] Gust J Hay KA Hanafi L-A Li D Myerson D Gonzalez-Cuyar LF . Endothelial activation and blood-brain barrier disruption in neurotoxicity after adoptive immunotherapy with CD19 CAR-T cells. Cancer Discov. (2017) 7:1404–19. doi: 10.1158/2159-8290.cd-17-0698 29025771 PMC5718945

[36] Shoaf ML Desjardins A . Oncolytic viral therapy for malignant glioma and their application in clinical practice. Neurotherapeutics. (2022) 19:1818–31. doi: 10.1007/s13311-022-01256-1 35674873 PMC9723031

[37] Pan P-W Haggiagi A . Neurologic immune-related adverse events associated with immune checkpoint inhibition. Curr Oncol Rep. (2019) 21:108. doi: 10.1007/s11912-019-0859-2 31776691

[38] Cuzzubbo S Javeri F Tissier M Roumi A Barlog C Doridam J . Neurological adverse events associated with immune checkpoint inhibitors: Review of the literature. Eur J Cancer. (2017) 73:1–8. doi: 10.1016/j.ejca.2016.12.001 28064139

[39] Schonfeld E Choi J Tran A Kim LH Lim M . The landscape of immune checkpoint inhibitor clinical trials in glioblastoma: A systematic review. Neuro-Oncol Adv. (2024) 6:vdae174. doi: 10.1093/noajnl/vdae174 39534539 PMC11555435

[40] Omuro A Vlahovic G Lim M Sahebjam S Baehring J Cloughesy T . Nivolumab with or without ipilimumab in patients with recurrent glioblastoma: Results from exploratory phase I cohorts of CheckMate 143. Neuro-Oncol. (2018) 20:674–86. doi: 10.1093/neuonc/nox208 29106665 PMC5892140

[41] Saha D Martuza RL Rabkin SD . Oncolytic herpes simplex virus immunovirotherapy in combination with immune checkpoint blockade to treat glioblastoma. Immunotherapy. (2018) 10:779–86. doi: 10.2217/imt-2018-0009 30008259 PMC6275562

[42] Majzner RG Theruvath JL Nellan A Heitzeneder S Cui Y Mount CW . CAR T cells targeting B7-H3, a pan-cancer antigen, demonstrate potent preclinical activity against pediatric solid tumors and brain tumors. Clin Cancer Res. (2019) 25(8):2560–74.. doi: 10.1158/1078-0432.ccr-18-0432 30655315 PMC8456711

[43] Vitanza NA Wilson AL Huang W Seidel K Brown C Gustafson JA . Intraventricular B7-H3 CAR T cells for diffuse intrinsic pontine glioma: Preliminary first-in-human bioactivity and safety. Cancer Discov. (2023) 13(1):114–31. doi: 10.1158/2159-8290.cd-22-0750 36259971 PMC9827115

[44] Rubin DB Danish HH Ali AB Li K LaRose S Monk AD . Neurological toxicities associated with chimeric antigen receptor T-cell therapy. Brain J Neurol. (2019) 142:1334–48. doi: 10.1093/brain/awz053 30891590

[45] Neelapu SS . Managing the toxicities of CAR T‐cell therapy. Hematol Oncol. (2019) 37(Suppl 1):48–52. doi: 10.1002/hon.2595 31187535

[46] Dubey D David WS Reynolds KL Chute DF Clement NF Cohen JV . Severe neurological toxicity of immune checkpoint inhibitors: Growing spectrum. Ann Neurol. (2020) 87:659–69. doi: 10.1002/ana.25708 32086972

[47] Hollingsworth RE Jansen K . Turning the corner on therapeutic cancer vaccines. NPJ Vaccines. (2019) 4:7. doi: 10.1038/s41541-019-0103-y 30774998 PMC6368616

[48] Ardon H Van Gool SW Lopes IS Maes W Sciot R Wilms G . Integration of autologous dendritic cell-based immunotherapy in the primary treatment for patients with newly diagnosed glioblastoma multiforme: A pilot study. J Neuro-Oncol. (2010) 99:261–72. doi: 10.1007/s11060-010-0131-y 20146084

