# Supplementary material for: Efficacy and safety of immunotherapy in pediatric malignant brain tumors: a systematic review and exploratory meta-analysis
Source: Front Oncol. 2026 Sep 25;16:1932372. doi: 10.3389/fonc.2026.1932372 (PMC13649463; doi:10.3389/fonc.2026.1932372)
Supplement: Supplementary file 1 [file Table1.docx]

Supplementary Table 1. Risk of Bias Assessment Using ROBINS-I and Certainty of Evidence (CoE) by Study

| **Study** | **Modality** | **Confounding** | **Selection** | **Intervention Classification** | **Deviations** | **Missing Data** | **Outcome Measurement** | **Reporting** | **Overall ROBINS-I** | **Certainty of Evidence** |
| --- | --- | --- | --- | --- | --- | --- | --- | --- | --- | --- |
| Monje 2024/25 | CAR-T (GD2) | Serious | Moderate | Low | Low | Moderate | Moderate | Moderate | **Serious** | Low |
| Lin 2024 | CAR-T (GD2-C7R) | Serious | Moderate | Low | Low | Moderate | Moderate | Moderate | **Serious** | Low |
| Vitanza 2023 | CAR-T (B7-H3) | Serious | Moderate | Low | Low | Moderate | Moderate | Moderate | **Serious** | Low |
| Vitanza 2025 | CAR-T (B7-H3) | Serious | Moderate | Low | Low | Moderate | Moderate | Moderate | **Serious** | Low |
| Bertrand 2024 | CAR-T (B7-H3) | Serious | Serious | Low | Low | Serious | Moderate | Serious | **Serious** | Very low |
| Vitanza 2021 | CAR-T (HER2) | Serious | Moderate | Low | Low | Moderate | Moderate | Moderate | **Serious** | Low |
| Gust 2025 | CAR-T (EGFR806) | Serious | Moderate | Low | Low | Moderate | Moderate | Moderate | **Serious** | Low |
| Khatua 2020 | Cellular (NK) | Serious | Moderate | Low | Low | Moderate | Moderate | Moderate | **Serious** | Low |
| Friedman 2021 | Oncolytic (HSV-1) | Moderate | Moderate | Low | Low | Low | Low | Moderate | **Moderate** | Low |
| Pérez-Larraya 2022 | Oncolytic (DNX-2401) | Moderate | Moderate | Low | Low | Low | Low | Moderate | **Moderate** | Moderate |
| Thompson 2023 | Oncolytic (PVSRIPO) | Serious | Moderate | Low | Low | Moderate | Moderate | Moderate | **Serious** | Low |
| Schuelke 2022 | Viral (Pelareorep) | Serious | Moderate | Low | Low | Moderate | Moderate | Moderate | **Serious** | Low |
| Yu 2025 | Oncolytic (MV-NIS) | Moderate | Moderate | Low | Low | Low | Low | Moderate | **Moderate** | Low |
| Dunkel 2023 (NIVO) | Checkpoint | Serious | Moderate | Low | Low | Moderate | Low | Moderate | **Serious** | Low |
| Dunkel 2023 (NIVO+IPI) | Checkpoint | Serious | Moderate | Low | Low | Moderate | Low | Moderate | **Serious** | Low |
| Dunkel 2023 (HGG) | Checkpoint | Serious | Moderate | Low | Low | Moderate | Low | Moderate | **Serious** | Low |
| Dunkel 2023 (MB) | Checkpoint | Serious | Moderate | Low | Low | Moderate | Low | Moderate | **Serious** | Low |
| Hwang PBTC-045 | Checkpoint | Serious | Serious | Low | Low | Moderate | Moderate | Serious | **Serious** | Very low |
| Johnson 2024 | Systemic | Serious | Moderate | Low | Low | Moderate | Moderate | Moderate | **Serious** | Low |
| Pollack 2014 | Vaccine | Moderate | Moderate | Low | Low | Low | Low | Low | **Moderate** | Moderate |
| Pollack 2016 (LGG) | Vaccine | Moderate | Moderate | Low | Low | Low | Low | Low | **Moderate** | Moderate |
| Pollack 2016 (MGG) | Vaccine | Moderate | Moderate | Low | Low | Low | Low | Low | **Moderate** | Moderate |
| Ardon 2010 | DC Vaccine | Moderate | Moderate | Low | Low | Low | Low | Moderate | **Moderate** | Moderate |

Supplementary Table 2. Summary of Risk of Bias and Certainty of Evidence by Outcome

| **Outcome** | **No. Studies** | **Overall ROBINS-I** | **Publication Bias** | **Inconsistency** | **Indirectness** | **Certainty of Evidence** |
| --- | --- | --- | --- | --- | --- | --- |
| Overall Survival (OS) | 23 | Moderate–Serious | Not detected | Serious | Serious | **Low** |
| 12-month OS (OS12) | 23 | Moderate–Serious | Not detected | Moderate | Serious | **Low** |
| Grade ≥3 Neurotoxicity | 15 | Moderate | Present | None (I² = 0%) | Low | **Moderate** |
