# Supplementary material for: Efficacy and safety of immunotherapy in pediatric malignant brain tumors: a systematic review and exploratory meta-analysis
Source: Front Oncol. 2026 Sep 25;16:1932372. doi: 10.3389/fonc.2026.1932372 (PMC13649463; doi:10.3389/fonc.2026.1932372)
Supplement: Supplementary file 2 [file Table2.docx]

**Supplementary Methods
Complete Database Search Strategies**

Search period: database inception through December 31, 2025.
Databases: PubMed/MEDLINE, Embase, and Scopus.
The search combined pediatric population terms, central nervous system tumor terms, and immunotherapy terms. No post-cutoff publication was eligible for inclusion in the quantitative synthesis.

**S1. PubMed/MEDLINE**

```
(
  ("Child"[Mesh] OR "Adolescent"[Mesh] OR pediatric*[Title/Abstract] OR paediatric*[Title/Abstract]
   OR child*[Title/Abstract] OR adolescen*[Title/Abstract])
  AND
  ("Brain Neoplasms"[Mesh] OR "Central Nervous System Neoplasms"[Mesh]
   OR brain tumor*[Title/Abstract] OR brain tumour*[Title/Abstract]
   OR CNS tumor*[Title/Abstract] OR CNS tumour*[Title/Abstract]
   OR glioma*[Title/Abstract] OR "high-grade glioma"[Title/Abstract]
   OR "high grade glioma"[Title/Abstract] OR HGG[Title/Abstract]
   OR "diffuse midline glioma"[Title/Abstract] OR DMG[Title/Abstract]
   OR "diffuse intrinsic pontine glioma"[Title/Abstract] OR DIPG[Title/Abstract]
   OR medulloblastoma*[Title/Abstract] OR ependymoma*[Title/Abstract])
  AND
  ("Immunotherapy"[Mesh] OR immunotherap*[Title/Abstract]
   OR "Chimeric Antigen Receptor T-Cell Therapy"[Mesh]
   OR CAR-T[Title/Abstract] OR "CAR T"[Title/Abstract] OR "CAR-T cell*"[Title/Abstract]
   OR "cellular therap*"[Title/Abstract] OR "NK cell*"[Title/Abstract]
   OR "natural killer cell*"[Title/Abstract]
   OR "Immune Checkpoint Inhibitors"[Mesh] OR "checkpoint inhibitor*"[Title/Abstract]
   OR nivolumab[Title/Abstract] OR pembrolizumab[Title/Abstract] OR ipilimumab[Title/Abstract]
   OR vaccine*[Title/Abstract] OR "dendritic cell vaccine*"[Title/Abstract]
   OR "peptide vaccine*"[Title/Abstract]
   OR "Oncolytic Virotherapy"[Mesh] OR "oncolytic virus*"[Title/Abstract]
   OR "oncolytic viral"[Title/Abstract] OR virotherap*[Title/Abstract]
   OR "viral therap*"[Title/Abstract])
)
```

**S2. Embase (Embase.com syntax)**

```
(
  ('child'/exp OR 'adolescent'/exp OR pediatric*:ti,ab OR paediatric*:ti,ab
   OR child*:ti,ab OR adolescen*:ti,ab)
  AND
  ('brain tumor'/exp OR 'central nervous system tumor'/exp
   OR 'brain tumor*':ti,ab OR 'brain tumour*':ti,ab
   OR 'cns tumor*':ti,ab OR 'cns tumour*':ti,ab
   OR glioma*:ti,ab OR 'high grade glioma':ti,ab OR hgg:ti,ab
   OR 'diffuse midline glioma':ti,ab OR dmg:ti,ab
   OR 'diffuse intrinsic pontine glioma':ti,ab OR dipg:ti,ab
   OR medulloblastoma*:ti,ab OR ependymoma*:ti,ab)
  AND
  ('immunotherapy'/exp OR immunotherap*:ti,ab
   OR 'chimeric antigen receptor T cell therapy'/exp
   OR 'car-t':ti,ab OR 'car t':ti,ab OR 'car-t cell*':ti,ab
   OR 'cellular therap*':ti,ab OR 'natural killer cell'/exp
   OR 'nk cell*':ti,ab OR 'natural killer cell*':ti,ab
   OR 'immune checkpoint inhibitor'/exp OR 'checkpoint inhibitor*':ti,ab
   OR nivolumab:ti,ab OR pembrolizumab:ti,ab OR ipilimumab:ti,ab
   OR 'cancer vaccine'/exp OR vaccine*:ti,ab
   OR 'dendritic cell vaccine*':ti,ab OR 'peptide vaccine*':ti,ab
   OR 'oncolytic virus'/exp OR 'oncolytic virus*':ti,ab
   OR 'oncolytic viral':ti,ab OR virotherap*:ti,ab OR 'viral therap*':ti,ab)
)
```

**S3. Scopus**

```
TITLE-ABS-KEY(
  (pediatric* OR paediatric* OR child* OR adolescen*)
  AND
  ("brain tumor*" OR "brain tumour*" OR "CNS tumor*" OR "CNS tumour*"
   OR glioma* OR "high-grade glioma" OR "high grade glioma" OR HGG
   OR "diffuse midline glioma" OR DMG
   OR "diffuse intrinsic pontine glioma" OR DIPG
   OR medulloblastoma* OR ependymoma*)
  AND
  (immunotherap* OR CAR-T OR "CAR T" OR "CAR-T cell*"
   OR "cellular therap*" OR "NK cell*" OR "natural killer cell*"
   OR "checkpoint inhibitor*" OR nivolumab OR pembrolizumab OR ipilimumab
   OR vaccine* OR "dendritic cell vaccine*" OR "peptide vaccine*"
   OR "oncolytic virus*" OR "oncolytic viral" OR virotherap* OR "viral therap*")
)
```

**S4. Study-selection procedure**

Search results were deduplicated before title and abstract screening. Title/abstract screening and full-text eligibility assessment were conducted by a single reviewer using predefined eligibility criteria. Data extraction was subsequently performed by the same single investigator using a predefined standardized form. The use of a single reviewer/investigator is acknowledged as a methodological limitation.

**S5. Reproducibility note**

The database-specific strings above should correspond exactly to the searches actually executed. If the original Embase search was run through Ovid rather than Embase.com, the Embase syntax should be replaced by the original Ovid search history before submission. Likewise, record counts should be reported from the original saved search histories rather than reconstructed retrospectively.
